# Optimal Geometrical Set for Automated Marker Placement to Virtualized Real-Time Facial Emotions

**DOI:** 10.1371/journal.pone.0149003

**Published:** 2016-02-09

**Authors:** Vasanthan Maruthapillai, Murugappan Murugappan

**Affiliations:** School of Mechatronic Engineering, Universiti Malaysia Perlis, 02600, Ulu Pauh, Arau, Perlis, West Malaysia; Universiti Malaysia Perlis, MALAYSIA

## Abstract

In recent years, real-time face recognition has been a major topic of interest in developing intelligent human-machine interaction systems. Over the past several decades, researchers have proposed different algorithms for facial expression recognition, but there has been little focus on detection in real-time scenarios. The present work proposes a new algorithmic method of automated marker placement used to classify six facial expressions: happiness, sadness, anger, fear, disgust, and surprise. Emotional facial expressions were captured using a webcam, while the proposed algorithm placed a set of eight virtual markers on each subject’s face. Facial feature extraction methods, including marker distance (distance between each marker to the center of the face) and change in marker distance (change in distance between the original and new marker positions), were used to extract three statistical features (mean, variance, and root mean square) from the real-time video sequence. The initial position of each marker was subjected to the optical flow algorithm for marker tracking with each emotional facial expression. Finally, the extracted statistical features were mapped into corresponding emotional facial expressions using two simple non-linear classifiers, K-nearest neighbor and probabilistic neural network. The results indicate that the proposed automated marker placement algorithm effectively placed eight virtual markers on each subject’s face and gave a maximum mean emotion classification rate of 96.94% using the probabilistic neural network.

## Introduction

Non-verbal communication plays an important role in developing intelligent machines that can exhibit better interaction with humans by closely emulating human-human communications. Researchers have increase their focus on developing an intelligent human-machine interface (HMI) system for assisting elderly people that could improve their quality of life [1, 2]. Human body gestures, postures, and facial expressions are used as non-verbal communication mediums to develop HMI systems. Among these modalities, facial expression is the most common due to its cost effectiveness, more reliable detection, and shorter computation time, among other advantages [2–5]. Over the past several decades, researchers have developed intelligent methodologies to effectively recognize human facial expressions that have been implemented in real-time systems for a variety of applications, such as video gaming, machine vision, pain assessment, psychology, behavioral analysis, and clinical diagnosis [6–9]. As a result, recent HMI systems can easily “understand” the expressions of humans and perform different tasks [10–12].

Emotions can be universally categorized into six types: anger, sadness, surprise, fear, happiness, and disgust. Emotions can be assessed using different modalities, such as physiological signals, gestures, speech, and facial expressions [5, 13–15]. Each method of emotion recognition has its own advantages and limitations. Although physiological signals inherently detect human emotions through either central and/or peripheral nervous system activities, issues with higher computational complexity, presence of noise and artifacts in acquired signals, and intrusive electrode placement on the human body limit the development of intelligent real-time systems. Furthermore, most subjects become uncomfortable wearing the electrodes all day long when interacting with systems for any given application. Indeed, most physiological signal-based emotion recognition systems have been developed within a controlled laboratory environment, and very few have been developed in real-time scenarios [16, 17]. Therefore, recent developments in novel image processing algorithms will likely make facial expression detection more reliable and effective for real-time system development over other modalities.

### Facial Action Coding System (FACS)

The Facial Action Coding System (FACS) was originally proposed by Ekman and Friesen [18, 19] to identify facial expression of human emotions. There are 46 AUs and 7000 proposed combinations for facial expression detection in the FACS. Although researchers have used different numbers of AUs for developing facial expression recognition systems in laboratory environments, very few have proposed the detection of facial expressions in real-time [20, 21]. Thus, no standard has been proposed for using either a specific set or combination of AUs to identify facial expression. Ekman and Friesen [18] previously discussed facial muscle activation with different emotions and defined the facial AU system for classification of facial expressions. S1 Table shows the effective changes of AUs in facial muscles for each emotion [22]. So, in this research FACS has been used as a guideline to identify the expressions. FACS acts as an investigative tool to study bout the movements of the markers when each expression take place. All eight virtual markers have been placed and investigate according to AU's.

### Face and Eye Detection

**Fig 1:**
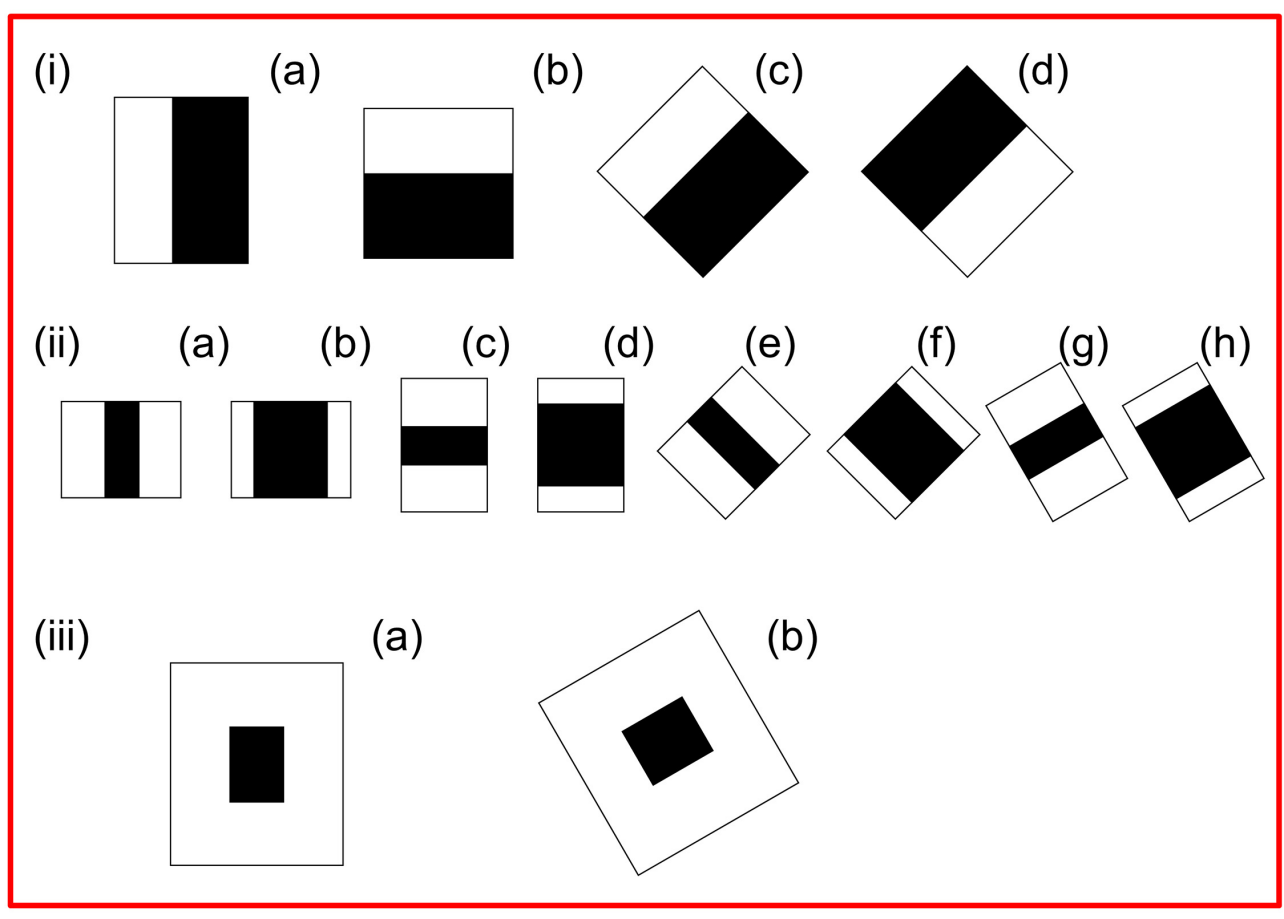
Common Haar Features; (i) Edge feature; (ii) Side features (iii) Centre-Surrounded Feature.

Face detection is a very important step in facial expression recognition. An efficient automated face detection system should be able to detect a subject’s face in complex scenes with cluttered backgrounds and be able to locate its exact position therein [23]. In face detection, facial features, such as the eyes, nose, and mouth, serve as reference points [24]. By scanning facial images at different resolutions, the scale or size of a face can be analyzed [25]. Several face detection methods have been reported for the recognition of facial expressions [11, 20]. However, AU-based face detection has been used more often in previous studies than other methods [4, 18]; such as, the Viola and Jones face detection method [26]. In real-time scenarios, face detection is performed through either image pixels or Haar-like features [27]. The image pixels-based approach requires a longer computation time to detect the face, and the number of pixels varies in proportion to face shape and pigmentation [28]. Haar-like features can be used to compute changes in pixel contrast (white and black) between adjacent rectangular groups instead of using original pixel values (Fig 1). A more detailed description of the Haar-like features method can be found in a previous study [29]. The Haar-like features method efficiently detects any object, including human faces, in a given image sequence using the AdaBoost cascade classifier [26]. The Viola and Jones method is used to detect faces using Haar-like features in a shorter time with less computational complexity [26]. S1 Fig shows the flow chart of the Viola and Jones algorithm for face detection.

In the current report, Haar-like features were used to detect the front of each subject’s face and their eyes. By using an Open CV library, the facial image captured by webcam was passed into the Open CV in order to detect faces via the Haar cascade classifier. Before sending to Open CV, the acquired facial image was subjected to double-precision formatting and converted to grayscale to reduce computational time and memory. Haar-like features in Open CV were used to detect each subject’s face. Haar-like classifiers can detect a subject’s face in 0.067 s compared to other face detection methods [26]. The system then creates an ellipse around the subject’s face and places a “+” mark on both eyes in order to position virtual markers on the subject’s face [30, 31]; most human faces are relatively ellipsoidal in shape. Hence, we drew the ellipse based on methods discussed previously [30, 31]. S2A Fig shows one subject’s facial image captured by webcam and S2B Fig shows the image after face and eye detection.

Therefore, in this research six basic emotions were classified. Haar like features are used to identify the user face and eye. A total of eight automated virtual markers is placed on user face at specific location which discussed in proposed method section. Previously this research examines with a total of ten, eight and six virtual markers to study the optimal number of markers which identify better emotion recognition. As a conclusion eight virtual markers gives better accuracy and based on previous study, eight virtual markers is the optimal number of marker [32]. Hence, in this paper eight virtual markers are discussed. All the markers are then mapped into optical flow algorithm (OFA) [33, 34] to predict the future point. The movement of the markers for each emotion, then investigate with the guide of FACS. The proper methods and the results are discussed clearly in the following sections.

## Proposed Method

The present work proposes a new method of automated virtual marker placement on a subject’s face that can be used to detect six basic emotional facial expressions and compares the emotion recognition performance of this new method with manual marker placement. Eight virtual markers were automatically placed at specific locations on each subject’s face, while a webcam captured emotional facial expression sequences. Initially, subjects were requested to place the markers manually on their face based on the given guidelines from the instructor. The guidelines are obtained from FACS, and marker positions were then used for developing an algorithm for automated marker placement. The flow of manual and automated marker placement methods for facial emotion detection is given in S3 Fig. Our complete algorithm was implemented in Microsoft Visual Studio with an Open CV library using C++ programming language on a desktop computer with an Intel i3 processor, 2 GB ROM, and Windows 8 operating system. The Haar cascade database in an Open Computer Vision (Open CV) library was used to detect each subject’s face from video sequences captured via webcam. Initial marker positions (x-y coordinates) were passed through the Lucas–Kanade OFA for predicting future marker positions. The distance of each marker from the center point of each subject’s face defined features for facial expression detection. Extracted features were then mapped with corresponding emotions using the two nonlinear classifiers K-nearest neighbor (KNN) and probabilistic neural network (PNN).

### Manual Marker Placement

**Fig 2:**
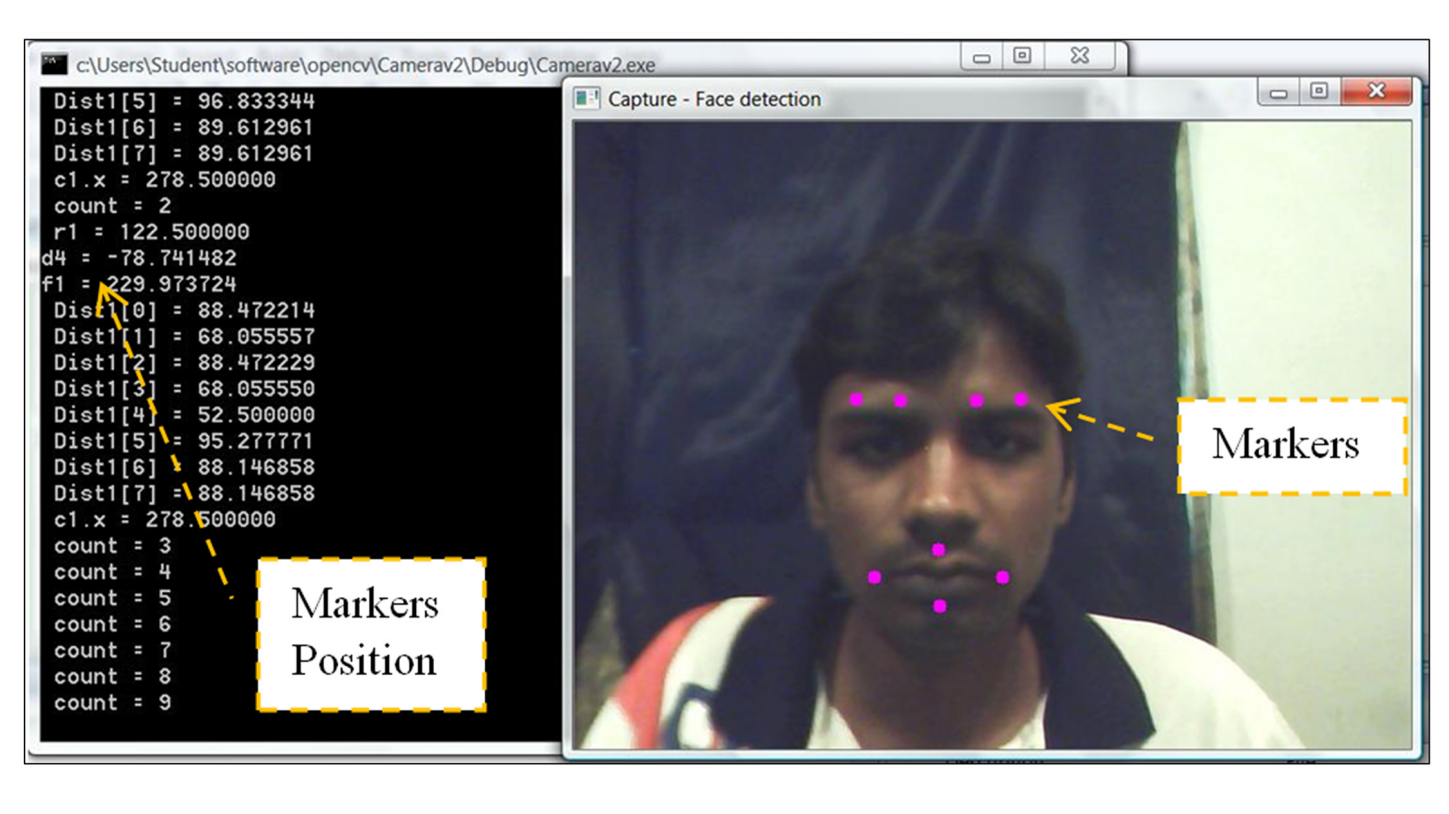
Markers placement and each marker's position; (Left side; the coordinate and distance calculating) (right side; all markers placed at specific position).

Manual marker placement was carried out to detect the mean position (distance between the center of the face to the marker’s location) of each marker on the subject’s face. This position was used to develop the automated marker placement algorithm for facial emotion recognition. In this experiment, subjects were requested to digitally place eight markers on their face in specified locations. The number of markers used for facial expression detection was devised by trial and error. The background was set with a black and white screen and the room light intensity was maintained at 41 lx. All subjects were seated comfortably on a chair placed in front of the computer monitor at a distance of 0.95 m. In total, 10 subjects with a mean (± standard deviation) age of 24 ± 0.84 years were assisted with manually placing the eight markers at defined locations on their facial image using the FACS [18]. Markers were placed by clicking the cursor at each position on the facial image. The system then automatically computed the center of the face [31], calculated each marker’s position, and subsequently saved the newly acquired information. Manually clicking the cursor at each of the eight defined facial positions allowed the system to record the exact x-y coordinates of each spot and insert a virtual marker (pink). Using the Pythagorean theorem, the distance between each marker and the center point of the face was calculated [31]. Each subject underwent three marker placement trials for each emotional facial expression, and the mean marker position distance was calculated with respect to the center of the face. S4 and S5 Figs show the experimental setup and manual marker placement on one subject and the marker position recorded by the system in Fig 2, respectively. Calculation of each marker position with reference to the center of the face via manual marker placement was then used to develop the automatic marker placement algorithm.

### Automatic Marker Placement

The eight virtual markers positioned according to the previous section were then used for automated facial expression detection, which is extremely convenient, computationally efficient (less computational time and memory), and works with the OFA for tracking markers. Liu *et al*. proposed a geometric facial model that created a rectangle around the subject’s face using eye positions [35]. The distance between the eyes is used to identify the center point of the face, followed by the mouth; this geometric model is shown in S6A Fig. Thus, the geometric facial model proposed by Liu *et al*. [35] was used to identify the center of each subject’s face by detecting the eyes. Herein, a total of eight markers were placed on the upper and lower face with reference to the center of the subject’s face; four markers each were placed on the upper face (two each on the left and right eyebrows) and lower face (one each above, below, to the left, and to the right of the mouth; S6B Fig). These marker positions were used for classifying the subject’s emotional facial expressions.

S6B Fig shows the placement of eight virtual markers (black) on a subject’s face from the center point. Initially, Haar-like features were used to detect the face and eyes of the subject. The system places a center marker after the computation of distance between two eyes. According to Liu *et al*. [35], the center point is located a quarter of the distance (in cm) from the eye to the mouth if the facial geometry is rectangular. In the current study, the mean marker position calculated after manual marker placement across 10 subjects was used for computing the center point of the subject’s face in the automated marker placement algorithm. Hence, half the distance (in cm) from the eye to mouth was used to position the center point for the entire subject’s in the study. An ellipse was then created around each subject’s face with reference to the center point. The radius of the ellipse was taken from the facial features previously detected by Haar cascade classifiers.

**Fig 3:**
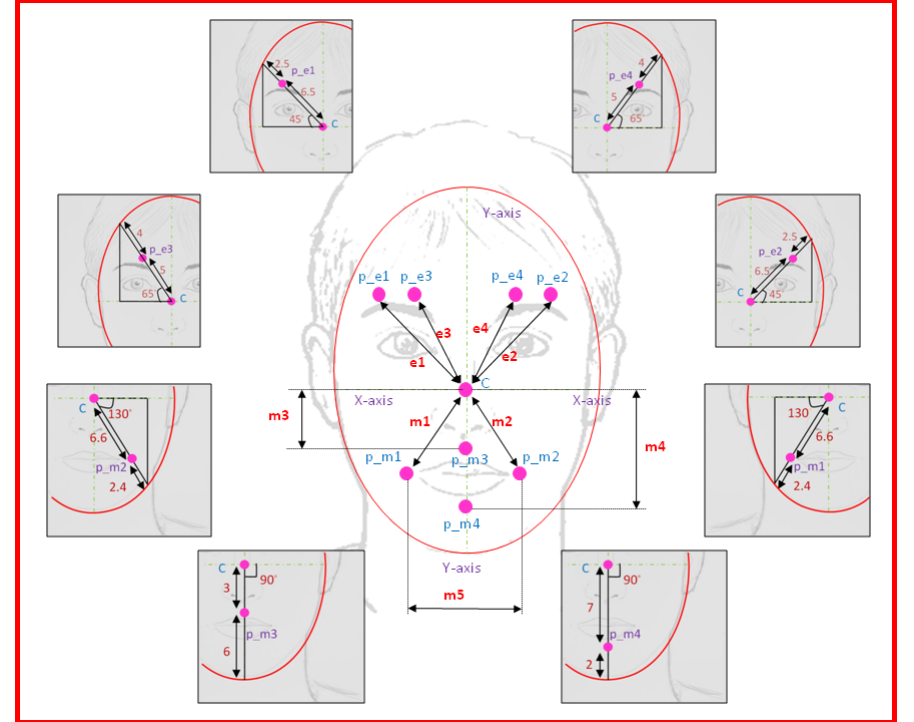
Marker Placement; The positions of upper face and lower face markers from center point with the respective angles and distance.

**Fig 4:**
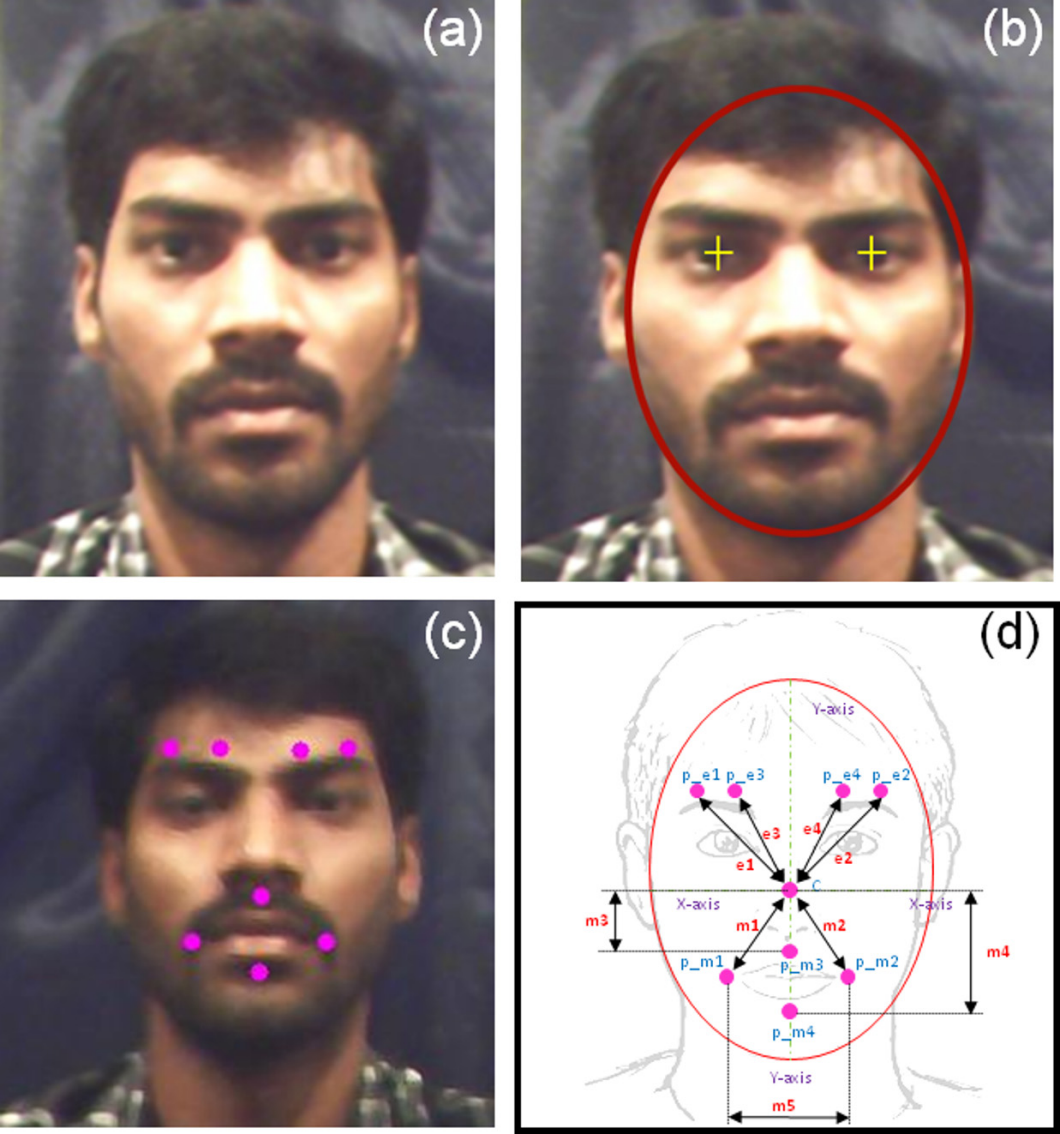
Automatic markers positions on the subject face; (a) user, (b) face and eye detection, (c) automated marker placement, (d) geometrical model of automated marker.

In general, facial shapes are not constant at all times; each face has its own shape (e.g., ellipsoidal, circular, etc.) and radius [36]. Thus, our new method uses ratios to calculate the radius of the ellipse. Initially, a vertical line was drawn from the center point to the intersection point between the ellipse and x-axis of the center point of the face (S6B Fig). Placement of the eight virtual markers was automatically done at a certain angle and distance from the center marker. The angle and distance (position) of each marker were computed from manual marker placement. The first and second markers of the upper face (p_e1 and p_e2) were placed at a 45° angle from the x-axis on the left and right sides of the face, respectively. Later, the radius of the ellipse at the 45° angle was calculated. From manual marker placement, it was determined that a mean marker distance ratio of 6.5:9 with the ellipse radius at a 45° angle provided the best positions for markers (p_e1 and p_e2) on the upper face. The second sets of markers (p_e3 and p_e4) on the upper face were placed at a 65° angle from the x-axis on the left and right sides, respectively. The mean distance ratio of the radius of the ellipse at an angle of 65° was found to be 5:9. The method used for upper face marker placement was then applied to the lower face. A complete description of the ratio calculation and placement of markers on the upper and lower face of each subject is given in Fig 3. Lower face markers were placed to the left, to the right, above, and below the subject’s mouth. Point’s p_m1 and p_m2 were placed at a 130° angle from the x-axis and had a mean distance ratio of 11:15; point’s p_m3 and p_m4 were fixed on the y-axis with mean distance ratios of 3:9 and 7:9, respectively. The placement of markers after the automated marker placement algorithm is shown in Fig 4.

The distance between the center point and each marker is referred to as a distance feature and considered an important feature of facial expression classification. A total of nine features were used: p_e1, p_e2, p_e3, p_e4, p_m1, p_m2, p_m3, p_m4, and p_m5. Eight features were the distance of each marker from center point, while the ninth (p_m5) was the distance between points to the left (p_m1) and right (p_m2) of the mouth (S7 Fig). In the current study, distance feature m1 was calculated using the Pythagorean theorem [31]. Every marker was assigned their own x-y coordinates [e.g., center point, (*x*_c_, *y*_c_); p_m1, (*x*_m1_, *y*_m1_)].

In Fig 3, in the left mouth column, line m1 is the hypotenuse of a right triangle, wherein the line parallel to the x-axis is *dx* [the difference between x-coordinates of the center point (*x*_c_) and p_m1 (*x*_m1_)]; and the line parallel to the y-axis is *dy* [the difference between y-coordinates of the center point (*y*_c_) and p_m1 (*y*_m1_)]. The formula for the computation of feature m1 is given in Eq (2):

$$Hypertenuse^{2}={\left(X_{c}-X_{m1}\right)}^{2}+{\left(Y_{c}-Y_{m1}\right)}^{2}$$ (1)

Therefore, the formula for feature m1 computation is given as in Eq (2):

$$\text{Feature distance}\;\left(\text{m}1\right)=\sqrt{{\left(X_{c}-X_{m1}\right)}^{2}+{\left(Y_{c}-Y_{m1}\right)}^{2}}$$ (2)

In a similar fashion, the distance of each marker from the center point was calculated using Eq 2. The coordinates of each marker were calculated using trigonometry formulas. The position of (x,y) each marker was found after calculating the feature distances at specific angles. Markers p_m3 and p_m4 were placed on the y-axis. Thus, their x-coordinates were the same as that of the center point, and their y-coordinates were found from the ratio of the y-axes. The coordinates of each feature were subjected to the OFA for future coordinate prediction. Initial coordinate values of each marker were replaced with future coordinate values for each marker, and the new distance from the center point was evaluated during facial expression. S2 Table presents the changes in distance of each marker for different emotions. Distance features e1, e2, e3, e4, m1, m2, m3, m4, and m5 indicate the initial position of the marker before facial expression, while features e1', e2', e3', e4', m1', m2', m3', m4', and m5' show the new position of each marker after facial expression.

## Results and Discussion

Facial expression recognition has been considered as a major research topic over the past several decades for developing intelligent systems [16, 17, 20, 37]. Most early work focused on AUs, and a little attention was paid to manual and virtual marker-based facial expression detection. AU-based facial expression detection is computationally intensive due to the large number and combination of AUs. On the other hand, manual marker placement is highly intrusive and subjects need not wear the markers (stickers) at all times. Indeed, this work demonstrated an automated lesser (8 marker) virtual marker placement. Most previous research has focused on recognizing six basic emotions (happiness, sadness, anger, fear, disgust, and surprise) through different numbers of AUs [20, 21, 38–42] were taken into the consideration.

### Data Collection for Emotion Recognition

**Fig 5:**
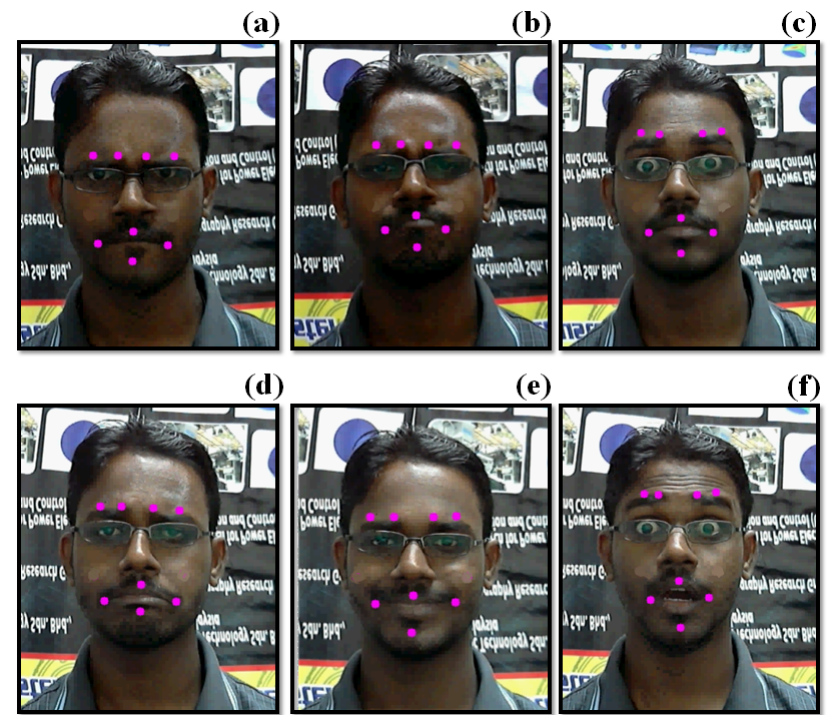
One subject’s emotional expressions with virtual markers; (a) anger, (b) disgust, (c) fear, (d) sadness, (e) happiness, (f) surprise.

The performance and reliability of emotion recognition systems are mainly based on data samples that are used to train the system. Participants provided written informed consent to participate in this study. The individual in this manuscript has given written informed consent to publish these case details. All research involving human participants have been approved by the Human Research Ethics Committee of Universiti Malaysia Perlis (HREC-UniMAP) and written consent has been obtained from the participants. In the present study, a sum of 30 subjects (14 male, 16 female) with a mean (± standard deviation) age range of (22.73 ± 1.68 years) were used to collect data on the six different emotional facial expressions in a video sequence. The subjects included in this study were of mixed ethnicities and religions (Hindu, Muslim, and Chinese) in Malaysia. All of the subjects were asked to express specific emotions following an instructor command in a controlled environment (lighting intensity: 41 lx; room temperature: 26°C; distance between the subject and camera: 0.95 m). The lighting intensity and distance between the subject and camera for this experiment were selected based on studies on different light intensities (3.00, 23.00, and 41.00 lx) and distances (near, 0.75 m; middle, 0.95 m; long, 1.15 m). Each emotional facial expression lasted 6 second and each expression was performed twice by each subject (two trials). Data collection was performed in a laboratory environment with two different backgrounds (one completely black in color, and another with a wall poster display). The total time required for completing the six emotional facial expressions (including instructions and baseline state) by one subject was 20 min. All subjects were healthy university students without any previous history of muscular, cognitive, or emotional disorders. Different emotional facial expressions for one subject with the eight virtual markers shown in Fig 5 and different subjects with the eight virtual markers are shown in S8 Fig.

Initially, manual marker placement on 10 subjects (triplicate) for each emotion was analyzed to identify the marker position (distance between the center of the face to each marker) on each subject’s face. Each subject was asked to place eight markers at defined facial locations based on the FACS. The mean value of each marker position with reference to the centre point and its position angle over 10 subjects were used to develop the automated marker placement algorithm and the results shown in Table 1. Different set of subjects was used for automated and manual marker placement. All the subjects are considered as unknown. Since a different ethnic group of subject was tested, different type of expression given by the subjects for each emotion. This issue was overcome by choosing eligible subjects and well explain the task to each subject.

**Table 1:** Manual marker position and its reading.

	Manual Marker Placement average of 3 trials															
Markers	Left eye_1(p_e1)		Left eye_2(p_e3)		Right eye_1(p_e2)		Right eye_2(p_e4)		Left mouth(p_m1)		Right mouth(p_m2)		Upper mouth(p_m3)		Lower mouth(p_m4)	
Subject	Angle in degree	Distance Ratio	Angle in degree	Distance Ratio	Angle in degree	Distance Ratio	Angle in degree	Distance Ratio	Angle in degree	Distance Ratio	Angle in degree	Distance Ratio	Angle in degree	Distance Ratio	Angle in degree	Distance Ratio
1	45.98	0.7623	66.13	0.7306	45.00	0.5742	66.16	0.5998	131.44	0.7348	130.03	0.7378	91.21	0.3582	90.65	0.3549
2	44.13	0.6985	64.67	0.7051	44.36	0.5298	64.50	0.5413	129.23	0.6989	129.54	0.6941	89.57	0.3173	89.25	0.3188
3	45.06	0.7374	66.00	0.7607	46.06	0.5673	66.03	0.5768	130.97	0.7368	130.63	0.7731	90.08	0.3548	91.02	0.3347
4	44.98	0.7042	64.05	0.6755	44.60	0.5358	64.01	0.5344	129.20	0.7230	129.26	0.6857	89.34	0.3267	89.67	0.3170
5	45.09	0.7495	65.72	0.7287	45.85	0.5910	65.16	0.5839	131.00	0.7610	130.05	0.7603	90.55	0.3598	90.73	0.3358
6	44.97	0.7114	64.77	0.6913	44.99	0.5519	64.92	0.5114	129.34	0.7261	129.42	0.7012	89.67	0.3153	89.41	0.2900
7	46.16	0.7334	65.37	0.7297	46.10	0.5581	65.79	0.6048	130.86	0.7400	130.20	0.7564	91.39	0.3333	90.54	0.3569
8	44.88	0.7087	64.44	0.6914	44.11	0.5215	64.96	0.5227	129.84	0.7284	129.65	0.7329	89.58	0.3327	89.02	0.2970
9	45.42	0.7280	65.45	0.7645	45.81	0.5840	65.95	0.5767	130.79	0.7439	131.07	0.7780	90.83	0.3683	90.03	0.3367
10	44.09	0.6983	64.42	0.7136	44.84	0.5076	64.36	0.5181	129.80	0.7027	129.89	0.7172	89.72	0.2831	89.58	0.2922
**Average**	**45.08**	**0.7232**	**65.10**	**0.7191**	**45.17**	**0.5521**	**65.18**	**0.5570**	**130.25**	**0.7296**	**129.97**	**0.7337**	**90.19**	**0.3350**	**89.99**	**0.3234**
**Std Dev**	0.670	0.022	0.726	0.029	0.731	0.028	0.765	0.035	0.850	0.019	0.559	0.033	0.745	0.026	0.703	0.025

### Evaluate Position of Automated Marker Placement

Table 1 shows the marker distance ratios and angles of three trials of six emotional expressions for 10 subjects using manual marker placement. As a result, the angle of deviation of left eye_1 and right eye_1 markers was approximately 45°, left eye_2 and right eye_2 markers was 65°, left mouth and right mouth markers was 130°, and upper and lower mouth markers was 90°. Similarly, marker distance ratios from the center point to the left eye, right eye, mouth, and above and below the mouth were approximately 0.72, 0.55, 0.73, and 0.33, respectively. These mean marker angles and distance ratios were used for marker positioning using the automated marker placement algorithm that was later used to evaluate marker positioning on the same 10 subjects who underwent manual marker placement. S3 Table shows the differences (error) in marker distance ratios and position angles between manual and automated marker placement algorithms. In some cases, the marker angle over eight markers gave an error value <0.05° and a distance ratio <0.2 between automated and manual marker placement methods (S3 Table). This indicates that automated marker placement successfully located markers on subject’s faces and effectively recognized emotional facial expressions. Hence, marker distance ratios and angles reported in Table 1 were used to develop and test our proposed emotional facial expression recognition system with a greater number of subjects.

### Evaluation with Classifier

Next, a new set of 30 subjects was recruited to test the six emotional facial expressions using the automated marker placement algorithm to develop a facial emotion recognition system. The same experimental setup described in manual marker placement was used to develop the facial expression recognition system. The proposed marker placement algorithm placed markers on each subject's face using distance ratios and position angles shown in Table 1. Each marker transferred its original position (x, y) to the OFA to trace future marker movement and direction. In any facial expression occurred immediately afte the automated marker placement method, then the new position of each marker for each emotion was saved by the system. The data were collected in real-time with automated marker placement, and facial expressions were recorded for every subject.

Most existing facial expression recognition systems in the literature perform offline analysis rather than real-time system development [37, 38]. In the current study, three simple statistical features [mean, root mean square (RMS), and variance] of marker distance (the distance between each marker to the center of the face) and changes in marker distance between the original (neutral) (between the original and new marker positions) and new marker position during emotional facial expression were extracted and normalized using binary and bipolar normalization methods [43]. Finally, these normalized features were fed into two nonlinear classifiers (KNN [44] and PNN [45]) to classify the emotional facial expressions. To accomplish this approach, a 10-fold cross-validation was used to segregate the training and testing data for facial expression classification [20]. In the KNN classifier, the value of K is varied from 2 to 10, and the value of K at 5 provided a higher mean emotion recognition rate; Euclidean distance was used as a distance measure. Therefore, only an emotion recognition rate with K = 5 was reported in the present study. In the PNN, the spread value (σ) varied from 0.01 to 0.1.

#### Marker distance (the distance between each marker to the center of the face) (MD)

The feature was extract from the distance of each marker from centre point. Table 2 and S4 Table shows the KNN and PNN classifier results for MD features respectively.

**Table 2:** Facial emotional expression recognition rate (in %) based on marker distance (MD) using KNN.

	WITHOUT NORMALIZE			WITH NORMALIZE					
				BINARY NORMALIZATION			BIPOLAR NORMALIZATION		
Features	Mean	RMS	Variance	Mean	RMS	Variance	Mean	RMS	Variance
Emotion									
**Anger**	93.33	89.17	45.00	87.50	85.00	45.83	85.83	87.50	47.50
**Disgust**	80.83	84.17	55.83	80.00	81.67	57.50	86.67	84.17	55.83
**Fear**	95.83	86.67	55.00	89.17	87.50	47.50	83.33	86.67	51.67
**Sadness**	76.67	85.83	49.17	89.17	84.17	50.00	91.67	85.00	43.33
**Happiness**	90.00	95.00	49.17	86.67	81.67	47.50	84.17	91.67	45.83
**Surprise**	90.00	93.33	63.33	90.83	94.17	51.67	92.50	91.67	55.83
**Average**	87.78	**89.03**	52.92	87.22	85.69	50.00	87.36	**87.78**	50.00
**Std Dev**	7.45	**4.33**	6.51	3.82	4.70	4.22	3.85	**3.23**	5.27

#### Changes in marker distance (small change of distance between the original and new marker positions) (CMD)

The feature will investigate the small changes of each marker when facial expression takes place. The system will calculate the distance moved by the marker and classify the rate of recognition. S5 Table and Table 3 shows the KNN and PNN classifier results for CMD features respectively.

**Table 3:** Facial emotional expression recognition rate (in %) based on changes in marker distance (CMD) using PNN.

	WITHOUT NORMALIZE			WITH NORMALIZE					
				BINARY NORMALIZE			BIPOLAR NORMALIZE		
Features	Mean	RMS	Variance	Mean	RMS	Variance	Mean	RMS	Variance
Emotion	sig = 0.02	sig = 0.02	sig = 0.02	sig = 0.08	sig = 0.07	sig = 0.01	sig = 0.09	sig = 0.09	sig = 0.01
Anger	100.00	100.00	81.67	79.17	87.50	39.17	69.17	98.33	54.17
Disgust	82.50	81.67	82.50	86.67	94.17	55.83	80.00	96.67	75.83
Fear	88.33	90.00	80.00	95.00	95.00	65.83	86.67	98.33	69.17
Sadness	81.67	74.17	78.33	90.83	82.50	47.50	89.17	89.17	56.67
Happiness	88.33	93.33	90.00	98.33	98.33	82.50	98.33	99.17	87.50
Surprise	90.00	93.33	78.33	98.33	100.00	45.83	97.50	100.00	62.50
**Average**	88.47	**88.75**	81.81	91.39	92.92	56.11	86.81	**96.94**	67.64
Std Dev	6.59	**9.31**	4.36	7.50	6.68	15.86	11.05	**3.97**	12.59

Based on current experimental results, the RMS feature of the changes in marker distance gave a slightly higher mean accuracy rate (96.94%) than the marker distance (96.81%) using the PNN. This result is likely due to the fact that changes in mean marker distance (i) effectively reflect the effect of different emotional expressions on selective markers compared to all markers used for analysis of marker distance, and (ii) measures subtle changes in marker position with each emotional expression. Research have previously analyzed marker distance for facial expression recognition and achieved a maximum mean classification rate of 94% using 19 facial features and the Random Forests classifier [46].

Herein, simple nonlinear classifiers KNN and PNN were used to classify emotional facial expressions. Statistical features extracted from two different methods of feature extraction and normalization (bipolar and binary) was used to map corresponding emotional expressions using the KNN and PNN. Bipolar normalization gave a slightly higher mean emotional expression classification rate (96.94%) compared with binary normalization (96.81%) and unnormalized data (93.83%) in the PNN. In the case of the KNN, binary normalization gave a higher mean emotional expression recognition rate (92.36%) than bipolar normalization (90.00%) and unnormalized data (91.39%).

The current experimental results indicate that the RMS gives a higher facial expression recognition rate compared to other statistical features (mean and variance). For both marker distance and changes in marker distance methods, the RMS gave maximum mean facial expression recognition rates of 96.94% and 96.81%, respectively; the mean feature performed better than the variance but worse than the RMS in emotional facial expression classification. In both methods of feature extraction, variance provided a much lower mean emotional facial expression recognition accuracy (81.81%). However, use of the variance feature with the KNN classifier performed better than the PNN on emotional facial expression recognition. Researchers have used these statistical features for emotional facial expression recognition [44, 46, 47] and shown that variance provided a lower mean facial expression recognition rate with raw data than other statistical features (i.e., mean and RMS) [46].

### Comparison

Table 4 shows a comparison of emotional facial expression classification from the present work with previous reports [20, 32, 37–40, 46, 48–51]. Most previous studies have used a greater number of facial features or AUs to classify emotional facial expressions. A maximum mean classification rate of 96.00% was achieved by classifying six emotional facial expressions using 26 facial features and the Random Forests classifier. The CK database [52] and geometric facial features were commonly used in earlier studies, and facial expression analysis was performed offline. The highest (122) and lowest (12) number of facial features used previously for facial expression classification achieved maximum mean accuracies of 88.83% and 85%, respectively. However, the present study proposed a new method of automated marker placement on the face of subject’s to classify six facial expressions and achieved a maximum mean classification rate of 96.94% using the RMS and PNN.

**Table 4:** Comparison of facial emotional expression classification (%) of this present work with earlier works.

	Earlier works												
Specification	Li Zhang et.al [37]	Bashir et.al [38]	Kotsia et.al [39]	P.Michel et.al [40]	M.SUK et.al [20]	Liconsol et.al [46]	Ghimire D. et.al [48]	Huang K, et.al [49]	Lincosol et.al [50]	Aliaa A. et.al [51]	Saeed A. et.al [32]	Present Work	
No of Markers	122	12	62	22	77	19	52	21–100	26	19	8	8	
Database	CK+ [52]	Own database (20 subjects)	CK+ [52]	CK+ [52]	CK+ [52]	Radboud facial database	CK+ [52]	JAFFE [54]	Radboud facial database	CK+ [52]	CK+ [52]	Own database (30 subjects)	Own database (30 subjects)
Method	FAU, Shape and Texture Feature Extraction	FAU, Reigen of interest (ROI) the movement of markers	FAU, Geometrical displacement extraction	FAU, feature displacement	FAU, displacement between current features and neutral features	FAU, displacement between current features and neutral features	geometric based features, and Adaboost	Optimal set of triangular facial features	FAU, geometric and appearance based features	Geometric and appearance based features, Appearance Feature	Geometric, image feature extraction, Colour and gradient information is used	FAU, geometric based features	FAU, geometric based features
Classifier	Neural Network	Guided Particle Swarm Optimization (GPSO) algorithm	SVM	SVM	SVM	KNN,SVM, Random Forests	SVM	Neural Network	Random Forests	Neural Network	SVM	KNN	PNN
Mean Accuracy (%)	88.83	85.00	94.00	86.00	85.80	94.00	95.17	95.65	96.00	95.66	83%	92.36	**96.64**

Most previous research has utilized virtual markers to analyze the movement of facial muscles during emotion recognition tasks [20, 21, 38–42]. Recently, virtual marker-based facial emotion recognition has become popular in addition to AUs [4]. Different sets of markers, from 12 to 62 [39], have been used to detect facial expressions in laboratory and real-time environments. In these works, virtual markers were placed on the subject’s face manually, and no automated marker placement procedure was reported. In contrast to AUs, virtual markers are highly flexible when investigating the movement of markers when facial expressions take place, convenient (no physical stickers/labels are worn on the face), and therefore, more suitable for real-time facial expression detection [38]. However, virtual marker-based face detection is affected by poor lighting (light intensities <30 lx) and camera pixel resolution. Virtual markers used in real-time applications are more stable if the lighting intensity is >30 lx and minimum camera pixel resolution is 640 × 480 [38]. Besides facial features, geometric features based emotion recognition is more popular in human expression detection [53].

In contrast to earlier studies, this present work requires fewer facial features and a simple feature extraction and classification method to classify emotional facial expressions in real-time with a higher recognition rate. Most of the earlier studies adopted manual marker placement and facial AU methods for identifying different facial expressions and very few studies have addressed real-time facial expression detection. Our proposed automated marker placement algorithm works effectively in real-time scenarios with less computational complexity (memory and computation time) than previously reported methods. However, it also has the following limitations: (i) emotional facial expression recognition was performed with a limited number of subjects. The accuracy of the emotion recognition rate might differ if our method were tested with more unknown subjects. (ii) The currently proposed algorithm should be tested with other international databases for standardization purposes. In the future, we hope to analyze different types of statistical features that more efficiently reflect subtle changes in marker position with each emotion to improve the mean classification accuracy. In addition, we intend to implement more intelligent statistical learning algorithms, such as SVM and ANN, to enhance the mean emotional facial expression classification rate.

## Conclusion

Facial expression recognition is an intense research topic with several applications. This paper presents a novel automated marker placement algorithm for emotional facial expression classification using marker distance ratios and angles. The lowest number of virtual marker (8 Marker) was placed in paticular places on a subject’s face in proposed automated manner to identify their emotional facial expressions. The OFA was used to track future positions of the markers during emotional expression. A simple set of statistical features were used for classifying the six different emotions tested (happiness, sadness, anger, fear, surprise, and disgust) using two nonlinear classifiers (KNN and PNN). The proposed automated marker placement method gives a maximum mean emotional facial expression recognition rate of 96.94% relative to earlier studies and the computational time approximately 0.3 seconds. Our proposed automated marker placement algorithm will be highly useful for developing intelligent diagnostics for clinical investigation and analysis of emotional behaviors in facial muscle disorder patients and other clinical situations. Furthermore, it may also prove to be very useful for developing of intelligent automated systems that can assist the elderly with HMI devices that enable communication with their surroundings.

## Supporting Information

S1 Fig Flow chart. Viola-Jones algorithm flow chart for face detection.
(DOCX)

S2 Fig Initial position of image capture. (A) Webcam image (B) Face and eye detection using Haar cascade classifiers.
(DOCX)

S3 Fig Flow chart of the study. (A) Flowchart of the manual marker placement (B): Flowchart of the automatic marker placement.
(DOCX)

S4 Fig Manual marker placement. Total of eight markers are placed manually.
(DOCX)

S5 Fig Experimental setup. A setup for data collection in manual marker placement.
(DOCX)

S6 Fig A geometrical model impliment; (A) Liu et.al geometrical model of the face; (B): Placement of markers based on geometric model.
(DOCX)

S7 Fig The positions of markers and features. Total of eight markers position with their specific names.
(DOCX)

S8 Fig Different subject emotional expressions with virtual markers. (A) anger, (B) disgust, (C) fear, (D) sadness, (E) happiness, (F) surprise.
(DOCX)

S1 Table Action Units studies by Ekman and Friesen. (DOCX)

S2 Table The changes of markers distance for each emotion. (DOCX)

S3 Table Error between manual and automated marker placement. (DOCX)

S4 Table Facial emotional expression recognition rate (in %) based on marker distance (MD) using PNN. (DOCX)

S5 Table Facial emotional expression recognition rate (in %) based on changes in marker distance (CMD) using KNN (K = 5). (DOCX)
