# Supplementary figures and images for: Optimal Geometrical Set for Automated Marker Placement to Virtualized Real-Time Facial Emotions
Source: PLoS One. 2016 Feb 9;11(2):e0149003. doi: 10.1371/journal.pone.0149003 (PMC4747560; doi:10.1371/journal.pone.0149003)

## S1 Fig.


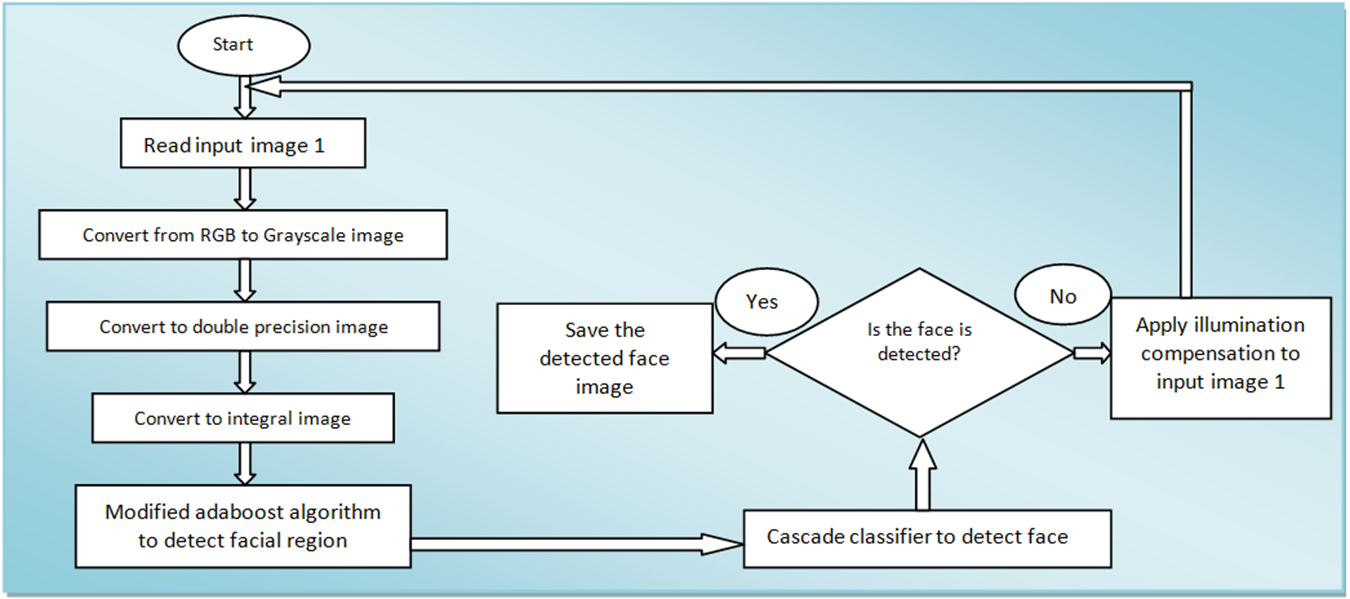

Supplement: S1 Fig — Viola-Jones algorithm flow chart for face detection. (DOCX) [file pone.0149003.s001.docx]

## S2 Fig.


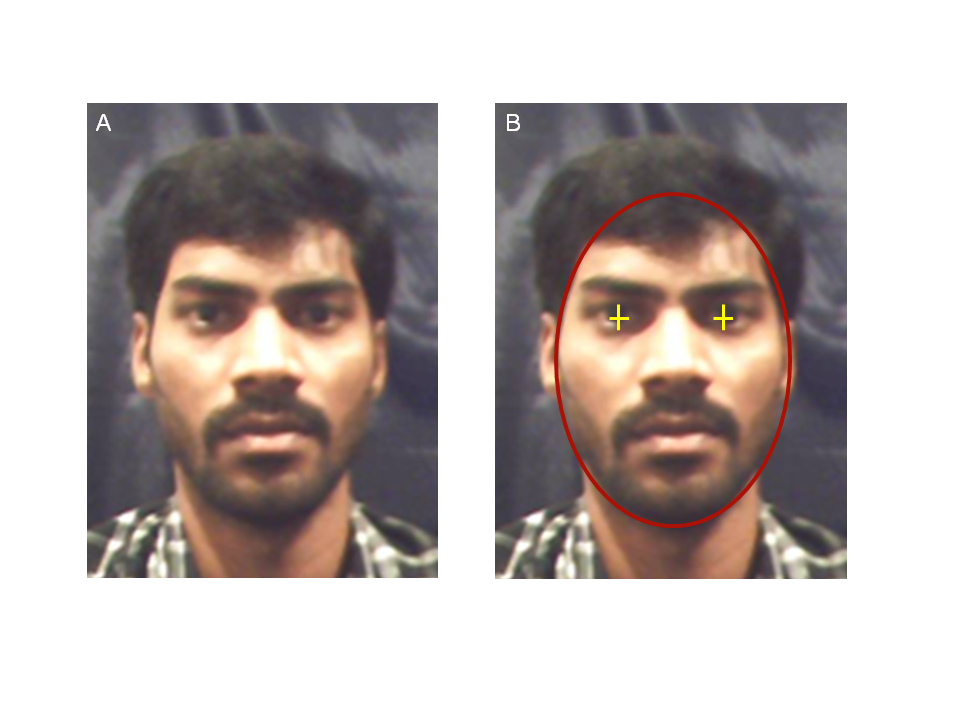

Supplement: S2 Fig — (A) Webcam image (B) Face and eye detection using Haar cascade classifiers. (DOCX) [file pone.0149003.s002.docx]

## S3 Fig.


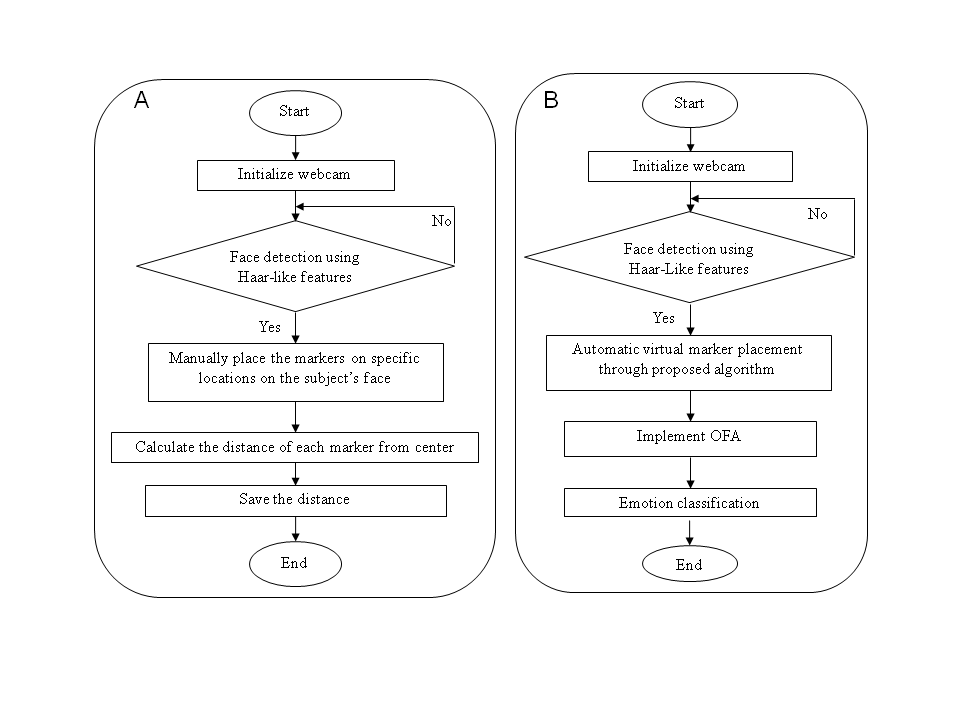

Supplement: S3 Fig — (A) Flowchart of the manual marker placement (B): Flowchart of the automatic marker placement. (DOCX) [file pone.0149003.s003.docx]

## S4 Fig.


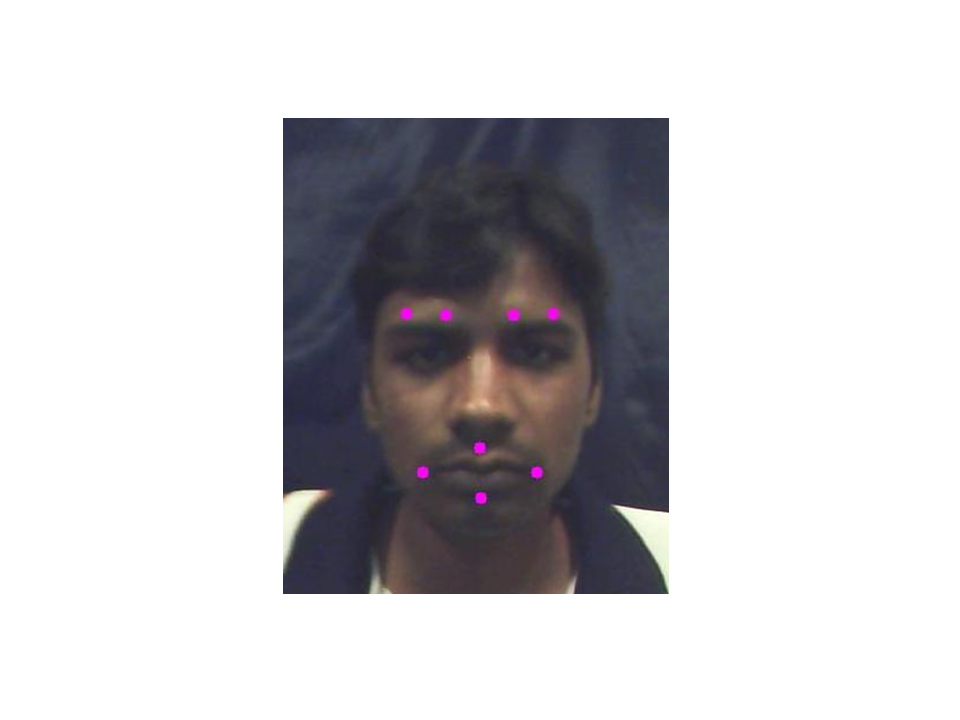

Supplement: S4 Fig — Total of eight markers are placed manually. (DOCX) [file pone.0149003.s004.docx]

## S5 Fig.


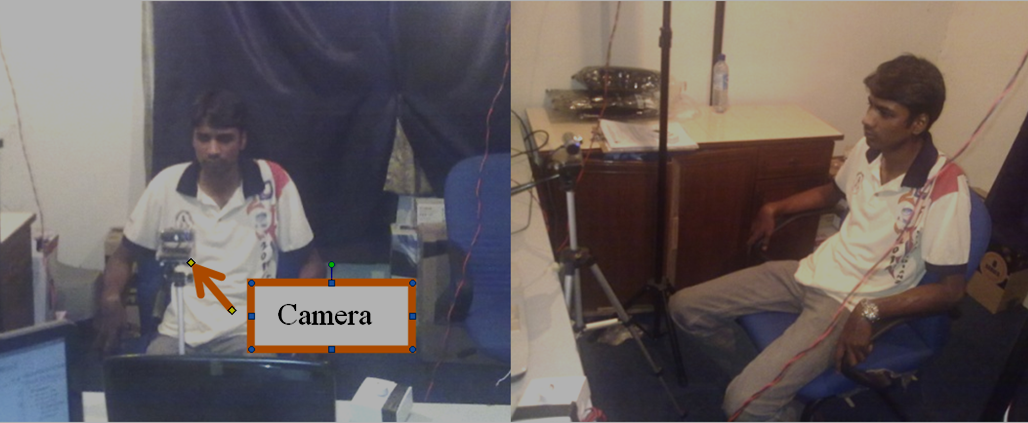

Supplement: S5 Fig — A setup for data collection in manual marker placement. (DOCX) [file pone.0149003.s005.docx]

## S6 Fig.


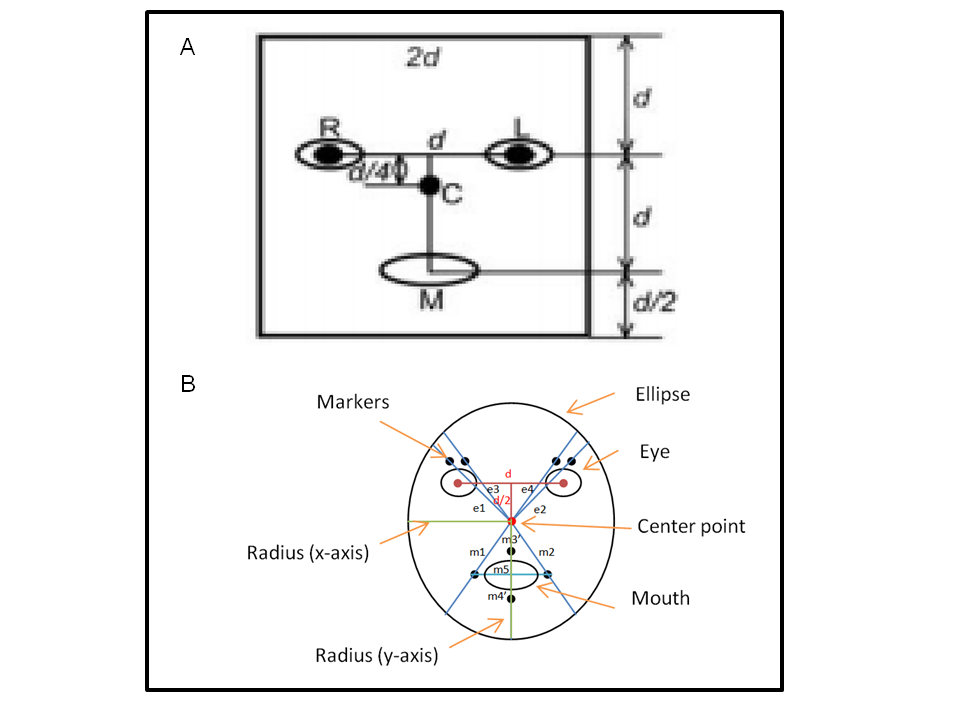

Supplement: S6 Fig — A geometrical model impliment; (A) Liu et.al geometrical model of the face; (B): Placement of markers based on geometric model. (DOCX) [file pone.0149003.s006.docx]

## S7 Fig.


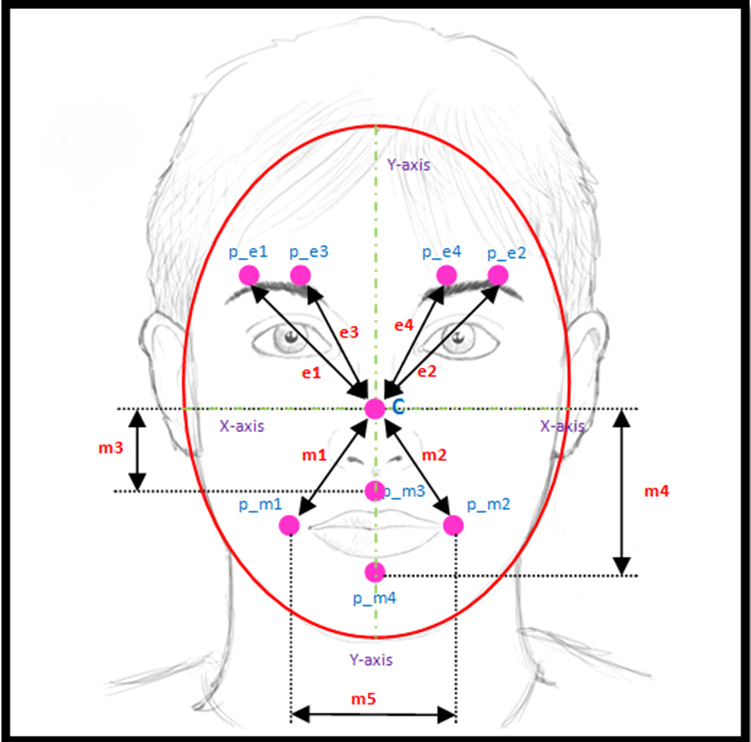

Supplement: S7 Fig — Total of eight markers position with their specific names. (DOCX) [file pone.0149003.s007.docx]

## S8 Fig.


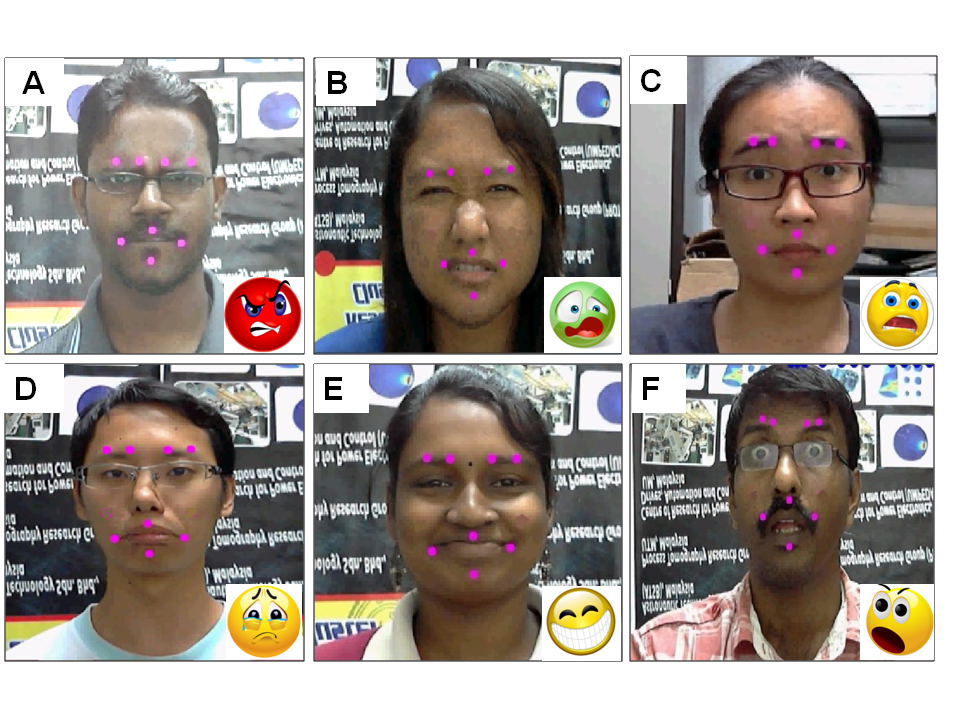

Supplement: S8 Fig — (A) anger, (B) disgust, (C) fear, (D) sadness, (E) happiness, (F) surprise. (DOCX) [file pone.0149003.s008.docx]
