# Supplementary material for: Optimal Geometrical Set for Automated Marker Placement to Virtualized Real-Time Facial Emotions
Source: PLoS One. 2016 Feb 9;11(2):e0149003. doi: 10.1371/journal.pone.0149003 (PMC4747560; doi:10.1371/journal.pone.0149003)
Supplement: S1 Table — (DOCX) [file pone.0149003.s009.docx]

## S1 Table

| **Emotions** | **Action Units (AU's)** | **FACS Name** | **Muscular Basis** |
| --- | --- | --- | --- |
| Happy | AU6 | Cheek Raiser | Orbicularis oculi (pars orbitalis) |
|  | AU12 | Lip Corner Puller | Zygomaticus major |
| Sad | AU14 | Dimpler | Buccinator |
|  | AU15 | Lip Corner Depressor | Depressor anguli Oris (also known as triangulation) |
| Surprise | AU12 | Lip Corner Puller | Zygomaticus major |
|  | AU5B | Upper Lid Raiser | Levator palpebrae superioris, superior tarsal muscle |
|  | AU26 | Jaw Drop | Masseter; relaxed temporalis and internal pterygoid |
| Fear | AU12 | Lip Corner Puller | Zygomaticus major |
|  | AU4 | Brow Lowerer | Depressor glabellae, depressor supercilii, corrugator supercilii |
|  | AU5 | Upper Lid Raiser | Levator palpebrae superioris, superior tarsal muscle |
|  | AU7 | Lid Tightener | Orbicularis oculi (pars palpebralis) |
|  | AU20 | Lip Stretcher | Risorius platysma |
|  | AU26 | Jaw Drop | Masseter; relaxed temporalis and internal pterygoid |
| Disgust | AU9 | Nose Wrinkler | Levator labii superioris alaeque nasi |
|  | AU15 | Lip Corner Depressor | Depressor anguli oris (also known as triangularis) |
|  | AU16 | Lower Lip | Depressor depressor labi inferioris |
| Anger | AU4 | Brow Lowerer | Depressor glabellae, depressor supercilii, corrugator supercilii |
|  | AU5 | Upper Lid Raiser | Levator palpebrae superioris, superior tarsal muscle |
|  | AU7 | Lid Tightener | Orbicularis oculi (pars palpebralis) |
|  | AU23 | Lip Tightener | Orbicularis oris |
