# Supplementary material for: Optimal Geometrical Set for Automated Marker Placement to Virtualized Real-Time Facial Emotions
Source: PLoS One. 2016 Feb 9;11(2):e0149003. doi: 10.1371/journal.pone.0149003 (PMC4747560; doi:10.1371/journal.pone.0149003)
Supplement: S2 Table — (DOCX) [file pone.0149003.s010.docx]

## S2 Table

| Feature | Distance between markers | Emotions | | | | | |
| --- | --- | --- | --- | --- | --- | --- | --- |
|  |  | Happiness | Sadness | Surprise | Fear | Anger | Disgust |
| e1 | C - p_e1 | e1 ≈ e1' | e1 ≈ e1' | e1 < e1' | e1 < e1' | e1 ≈ e1' | e1 > e1' |
| e2 | C - p_e2 | e2 ≈ e2' | e2 ≈ e2' | e2 < e2' | e2 < e2' | e2 ≈ e2' | e2 > e2' |
| e3 | C - p_e3 | e3 ≈ e3' | e3 ≈ e3' | e3 < e3' | e3 < e3' | e3 > e3' | e3 > e3' |
| e4 | C - p_e4 | e4 ≈ e4' | e4 ≈ e4' | e4 < e4' | e4 < e4' | e4 > e4' | e4 > e4' |
| m1 | C - p_m1 | m1< m1' | m1< m1' | m1 < m1' | m1 ≈ m1' | m1 ≈ m1' | m1 > m1' |
| m2 | C - p_m2 | m2 < m2' | m2 < m2' | m2 < m2' | m2 ≈ m2' | m2 ≈ m2' | m2 > m2' |
| m3 | C - p_m3 | m3 ≈ m3' | m3 ≈ m3' | m3 < m3' | m3 ≈ m3' | m3 ≈ m3' | m3 ≈ m3' |
| m4 | C - p_m4 | m4 ≈ m4' | m4 > m4' | m4 < m4' | m4 ≈ m4' | m4 > m4' | m4 > m4' |
| m5 | p_m1 - p_m2 | m5 < m5' | m5 ≈ m5' | m5 > m5' | m5 ≈ m5' | m5 > m5' | m5 ≈ m5' |

* C= Center marker position

* ' = new marker position after expression
