# Supplementary material for: Optimal Geometrical Set for Automated Marker Placement to Virtualized Real-Time Facial Emotions
Source: PLoS One. 2016 Feb 9;11(2):e0149003. doi: 10.1371/journal.pone.0149003 (PMC4747560; doi:10.1371/journal.pone.0149003)
Supplement: S3 Table — (DOCX) [file pone.0149003.s011.docx]

## S3 Table

|  | **Manual Marker Placement Error with 3 trials** | | | | | | | | | | | | | | | |
| --- | --- | --- | --- | --- | --- | --- | --- | --- | --- | --- | --- | --- | --- | --- | --- | --- |
| Markers  Subject | Left eye_1 (p_e1) | | Left eye_2 (p_e3) | | Right eye_1 (p_e2) | | Right eye_2 (p_e4) | | Left mouth (p_m1) | | Right mouth (p_m2) | | Upper mouth (p_m3) | | Lower mouth (p_m4) | |
|  | Angle in degree | Distance Ratio | Angle in degree | Distance Ratio | Angle in degree | Distance Ratio | Angle in degree | Distance Ratio | Angle in degree | Distance Ratio | Angle in degree | Distance Ratio | Angle in degree | Distance Ratio | Angle in degree | Distance Ratio |
| 1 | 0.049 | 0.230 | 0.094 | 0.059 | 0.010 | 0.180 | 0.099 | 0.050 | 0.030 | 0.004 | 0.065 | 0.028 | 0.025 | 0.107 | 0.059 | 0.103 |
| 2 | 0.004 | 0.156 | 0.037 | 0.184 | 0.026 | 0.139 | 0.006 | 0.119 | 0.007 | 0.145 | 0.093 | 0.256 | 0.097 | 0.006 | 0.035 | 0.254 |
| 3 | 0.051 | 0.260 | 0.075 | 0.242 | 0.025 | 0.290 | 0.058 | 0.047 | 0.022 | 0.048 | 0.006 | 0.145 | 0.038 | 0.188 | 0.015 | 0.163 |
| 4 | 0.026 | 0.172 | 0.037 | 0.023 | 0.070 | 0.145 | 0.046 | 0.208 | 0.087 | 0.180 | 0.028 | 0.101 | 0.038 | 0.131 | 0.071 | 0.190 |
| 5 | 0.030 | 0.141 | 0.006 | 0.106 | 0.048 | 0.256 | 0.057 | 0.010 | 0.084 | 0.106 | 0.033 | 0.232 | 0.031 | 0.138 | 0.027 | 0.260 |
| 6 | 0.029 | 0.185 | 0.098 | 0.113 | 0.069 | 0.171 | 0.099 | 0.200 | 0.059 | 0.094 | 0.061 | 0.214 | 0.068 | 0.229 | 0.035 | 0.086 |
| 7 | 0.058 | 0.209 | 0.050 | 0.205 | 0.014 | 0.224 | 0.024 | 0.291 | 0.010 | 0.039 | 0.097 | 0.183 | 0.001 | 0.190 | 0.058 | 0.253 |
| 8 | 0.009 | 0.157 | 0.088 | 0.218 | 0.094 | 0.169 | 0.081 | 0.009 | 0.011 | 0.084 | 0.012 | 0.038 | 0.057 | 0.238 | 0.074 | 0.095 |
| 9 | 0.031 | 0.146 | 0.017 | 0.079 | 0.092 | 0.273 | 0.004 | 0.277 | 0.045 | 0.258 | 0.076 | 0.168 | 0.058 | 0.015 | 0.027 | 0.004 |
| 10 | 0.093 | 0.288 | 0.030 | 0.178 | 0.062 | 0.267 | 0.079 | 0.280 | 0.042 | 0.089 | 0.065 | 0.262 | 0.056 | 0.219 | 0.030 | 0.135 |
| **Average** | **0.038** | **0.194** | **0.053** | **0.141** | **0.051** | **0.211** | **0.055** | **0.149** | **0.040** | **0.105** | **0.053** | **0.163** | **0.047** | **0.146** | **0.043** | **0.154** |
| **Std Dev** | 0.026 | 0.051 | 0.033 | 0.074 | 0.031 | 0.057 | 0.035 | 0.115 | 0.029 | 0.074 | 0.032 | 0.085 | 0.026 | 0.084 | 0.021 | 0.086 |
