# Supplementary material for: Optimal Geometrical Set for Automated Marker Placement to Virtualized Real-Time Facial Emotions
Source: PLoS One. 2016 Feb 9;11(2):e0149003. doi: 10.1371/journal.pone.0149003 (PMC4747560; doi:10.1371/journal.pone.0149003)
Supplement: S4 Table — (DOCX) [file pone.0149003.s012.docx]

## S4 Table

|  | **WITHOUT NORMALIZE** | | | **WITH NORMALIZE** | | | | | |
| --- | --- | --- | --- | --- | --- | --- | --- | --- | --- |
|  |  | | | **BINARY NORMALIZATION** | | | **BIPOLAR NORMALIZATION** | | |
| Features | Mean | RMS | Variance | Mean | RMS | Variance | Mean | RMS | Variance |
| Emotion | sig =0.02 | sig =0.02 | sig =0.01 | sig=0.05 | sig =0.05 | sig =0.01 | sig=0.10 | sig=0.09 | sig=0.01 |
| Anger | 100.00 | 100.00 | 84.17 | 86.67 | 89.17 | 35.83 | 90.83 | 93.33 | 64.17 |
| Disgust | 93.33 | 85.00 | 92.50 | 95.83 | 95.83 | 62.50 | 91.67 | 95.83 | 77.50 |
| Fear | 93.33 | 90.00 | 90.00 | 92.50 | 92.50 | 64.17 | 92.50 | 97.50 | 65.83 |
| Sadness | 90.00 | 98.33 | 84.17 | 95.00 | 95.00 | 50.83 | 95.00 | 97.50 | 62.50 |
| Happiness | 100.00 | 93.33 | 91.67 | 95.83 | 97.50 | 83.33 | 98.33 | 98.33 | 84.17 |
| Surprise | 93.33 | 96.67 | 90.83 | 96.67 | 96.67 | 45.00 | 96.67 | 98.33 | 65.83 |
| **Average** | **93.83** | **93.33** | 88.89 | 93.75 | 94.44 | 56.94 | 94.17 | **96.81** | 70.00 |
| Std Dev | **2.46** | **5.06** | 3.75 | 3.75 | 3.10 | 16.76 | 2.98 | **1.93** | 8.74 |
