# Supplementary material for: Optimal Geometrical Set for Automated Marker Placement to Virtualized Real-Time Facial Emotions
Source: PLoS One. 2016 Feb 9;11(2):e0149003. doi: 10.1371/journal.pone.0149003 (PMC4747560; doi:10.1371/journal.pone.0149003)
Supplement: S5 Table — (DOCX) [file pone.0149003.s013.docx]

## S5 Table

|  | **WITHOUT NORMALIZE** | | | **WITH NORMALIZE** | | | | | |
| --- | --- | --- | --- | --- | --- | --- | --- | --- | --- |
|  |  | | | **BINARY NORMALIZATION** | | | **BIPOLAR NORMALIZATION** | | |
| **Features**  **Emotion** | Mean | RMS | Variance | Mean | RMS | Variance | Mean | RMS | Variance |
| **Anger** | 84.17 | 87.50 | 45.83 | 91.67 | 92.50 | 40.83 | 87.50 | 86.67 | 39.17 |
| **Disgust** | 90.00 | 80.00 | 54.17 | 85.83 | 80.00 | 52.50 | 85.83 | 82.50 | 52.50 |
| **Fear** | 91.67 | 95.83 | 57.50 | 93.33 | 89.17 | 53.33 | 94.17 | 87.50 | 54.17 |
| **Sadness** | 91.67 | 85.00 | 47.50 | 89.17 | 81.67 | 45.83 | 87.50 | 82.50 | 45.00 |
| **Happiness** | 93.33 | 96.67 | 46.67 | 95.83 | 94.17 | 48.33 | 92.50 | 90.83 | 47.50 |
| **Surprise** | 97.50 | 92.50 | 59.17 | 98.33 | 96.67 | 59.17 | 92.50 | 92.50 | 53.33 |
| **Average** | **91.39** | 89.58 | 51.81 | **92.36** | 89.03 | 50.00 | 90.00 | 87.08 | 48.61 |
| **Std Dev** | **4.37** | 6.56 | 5.88 | **4.52** | 6.82 | 6.41 | 3.46 | 4.14 | 5.86 |
